# Genetic analysis and morphological identification of pilus-like structures in members of the genus *Bifidobacterium*

**DOI:** 10.1186/1475-2859-10-S1-S16

**Published:** 2011-08-30

**Authors:** Elena Foroni, Fausta Serafini, Davide Amidani, Francesca Turroni, Fei He, Francesca Bottacini, Mary O’Connell Motherway, Alice Viappiani, Ziding Zhang, Claudio Rivetti, Douwe van Sinderen, Marco Ventura

**Affiliations:** 1 Laboratory of Probiogenomics, Department of Genetics, Biology of Microorganisms, Anthropology and Evolution, University of Parma, Italy; 2 Dipartimento di Biochimica e Biologia Molecolare, University of Parma, Italy; 3 State Key Laboratory of Agrobiotechnology, College of Biological Sciences, China Agricultural University, Beijing 100193, China; 4 Alimentary Pharmabiotic Centre and Department of Microbiology, Bioscience Institute, National University of Ireland, Western Road, Cork, Ireland

## Abstract

**Background:**

Cell surface pili in Gram positive bacteria have been reported to orchestrate the colonization of host tissues, evasion of immunity and the development of biofilms. So far, little if any information is available on the presence of pilus-like structures in human gut commensals like bifidobacteria.

**Results and discussion:**

In this report, Atomic Force Microscopy (AFM) of various bifidobacterial strains belonging to *Bifidobacterium bifidum*, *Bifidobacterium longum* subsp. *longum*, *Bifidobacterium dentium*, *Bifidobacterium adolescentis* and *Bifidobacterium animalis* subsp. *lactis* revealed the existence of appendages resembling pilus-like structures. Interestingly, these microorganisms harbour two to six predicted pilus gene clusters in their genome, with each organized in an operon encompassing the major pilin subunit-encoding gene (designated *fim*A or *fim*P) together with one or two minor pilin subunit-encoding genes (designated as *fim*B and/or *fim*Q), and a gene encoding a sortase enzyme (*str*A). Quantitative Real Time (qRT)-PCR analysis and RT-PCR experiments revealed a polycistronic mRNA, encompassing the *fim*A/P and *fim*B/Q genes, which are differentially expressed upon cultivation of bifidobacteria on various glycans.

## Introduction

Pili or fimbriae are hair-like appendages commonly found in various Gram negative and Gram positive bacteria (for a review see [1]). Unlike Gram negative pili, whose subunits associate via non-covalent interactions, most pili detected in Gram positive bacteria are formed by covalent polymerization of pilin subunits, orchestrated by transpeptidase enzymes called sortases [2,3]. The general principles of pilus assembly/structure in Gram positive bacteria were first established for the pathogens *Corynebacterium diphtheriae* and *Actinomyces naeslundii*[2-5]. In these microorganisms, the genes for pilus formation and assembly are arranged in pathogenic islets that each encode one major pilin (represented by either SpaA, SpaD, or SpaH in *C.diphtheriae*, or FimA or FimP in *A. naeslundii*; Spa stands for sortase-mediated pilus assembly and Fim for fimbria-associated adhesion, respectively), one or two minor pilins (represented by the SpaB and SpaC, SpaE and SpaF, or SpaG and SpaI subunit couples in *C.diphtheriae*, or the FimB or FimQ subunits in *A. naeslundii*), and a pilus-specific sortase) ([1,2,4,6,7]. In *Actinomyces* the *fim*QP and the *fim*AB-loci encode fimbrial structures composed of a shaft protein, being either FimA or FimP, and a tip protein, represented by either FimB or FimQ, respectively [3,8]. Pilus gene clusters that encode a sortase have been found in many important Gram positive pathogens including *Clostridium perfringens*, enterococci and various streptococcal species, as well as in the *Actinomyces* taxon [1,2,7,9]. Recently, two pilus gene clusters have been identified in the genome of a human gut commensal *Lactobacillus rhamnosus* GG [10,11].

It has been shown that pili are involved in the attachment/colonization of members of the human microbiota to host tissues, as pilus-mediated adherence is a critical step in the establishment of infection by several Gram negative pathogens [12][13] as well as in Gram positive bacteria like corynebacteria [14] and *Actinomyces*[8]. In *Enterococcus faecalis*, pili are needed for biofilm production and non-piliated mutants are dramatically attenuated in an endocarditis model [9]. Furthermore, the presence of pili in streptococci was demonstrated to stimulate the host inflammatory response [13]. Recently, the mucosal adhesion features of the human intestinal bacterium, *Lactobacillus rhamnosus* GG were demonstrated to be due to the SpaCBA and SpaFED pilin subunits encoded by this organism [11].

Bifidobacteria are the most numerous bacterial members of the gut microbiota of infants [15,16]. They have been claimed to elicit several health-promoting or probiotic effects, such as strengthening of the intestinal barrier, modulation of the immune response and exclusion of pathogens [17,18]. Although there is some evidence to support each of these functional claims for particular bifidobacterial strains, the molecular mechanisms by which these activities are achieved remain largely unknown. Genome sequencing efforts have started to highlight the genetic strategies followed by bifidobacteria in order to colonize the human gut [19-23]. In a recent study, we discovered that the genome of *Bifidobacterium bifidum* PRL2010 harbors a large gene set involved in the utilization of host-derived glycans, such as those found in the outermost layer of the intestinal mucosa [24]. These findings therefore represent a clear example of host-microbe co-evolution, and presented *B. bifidum* PRL2010 as a bifidobacterial prototype for the analysis of the interaction between microbes and the intestinal mucosa.

The current study provides the first morphological evidence of pili-like structures decorating the cell surface of various bifidobacterial species. It further investigates the genetic organization and transcriptional profiling of the presumed bifidobacterial pilus encoding-gene clusters in response to different growth substrates.

## Material and methods

### Bacterial strains and culture conditions

Bifidobacterial cultures were incubated in an anaerobic atmosphere (2.99 % H_2_, 17.01 % CO_2_ and 80 % N_2_) in a chamber (Concept 400, Ruskin) in the Man-Rogosa-Sharp (MRS) (Scharlau Chemie, Barcelona, Spain) supplemented with 0.05 % (w/v) L-cysteine hydrochloride and incubated at 37°C for 16 h. Bifidobacterial cells were also cultivated on MRS where glucose was replaced with 2% (w/v) of an alternative carbon source (lactose, Fructo Oligosaccharides [FOS], mucin or N-acetyl glucosamine).

### Sample preparation and AFM imaging

Bacteria from four ml of a bacterial culture were harvested by centrifugation at 4000 rpm and resuspended in 200 µl of PBS (or 20 mM Hepes 7.5, 1 mM EDTA). 200 µl of 5% glutaraldehyde was added, followed by gentle mixing and incubation for 1 minute at room temperature. Thereafter, bacteria were washed four times with PBS by repeated resuspension and collection by centrifugation (4000 rpm). The washed pellet was then resuspended in 200 µl of PBS and kept on ice until AFM imaging.

To facilitate adhesion of bacteria to the mica support used for AFM imaging, mica was coated with polylysine (PL) as follows: 10 µl of a polylysine solution (10 ng/ml) was deposited onto freshly-cleaved mica for one minute. Mica was then rinsed with milliQ water (Millipore) and dried with nitrogen. After this, 20 µl of bacterial suspension was deposited onto PL-coated mica for 2-5 minutes depending on the particular strain or specific cultivation conditions. The mica disk was then rinsed with milliQ water and dried under a weak gas flow of nitrogen. Quality of the sample and density of surface-bound bacteria were verified with an optical microscope.

AFM imaging was performed on dried samples with a Nanoscope III microscope (Digital Instruments) equipped with scanner J and operating in tapping mode. Commercial diving board silicon cantilevers (MikroMasch) were used. Best image quality was obtained with high driving amplitude (1-3V) and low scan rate (0.5 Hz). Filamentous structures at the periphery of bacteria were visible in images of 512 x 512 pixels, representing a scansize of 10 µm or less. While imaging both height and amplitude signals were collected. Height images were flattened using Gwyddion software.

### Phylogenetic analyses

Genomic survey of the pili-encoding genes were performed by BLAST analysis against the NCBI database of the bifidobacterial FimA/P (cutoff: E-value 1 x 10-4 and 30% identity over at least 80% of both protein sequences).

Phylogeny calculations, including distance calculations and the generation of phylogenetic trees, were performed using PHYLIP (Phylogeny Inference Package) [25] version 3.5c. Trees were calculated using the neighbour-joining method under Kimura’s two-parameter substitution model [26]. Bootstrap values were computed by performing 1000 re-samplings. Dendograms from gene sequences were drawn using the ClustalW program (http://www.ebi.ac.uk/Tools/clustalw2/index.html) and were visualized with the TreeView program (http://taxonomy.zoology.gla.ac.uk/rod/treeview.html).

### 3D structure prediction of pilin major subunit

The 3D structures of pilin major subunits encoded by the so-called *pil* loci of *B. bifidum* PRL2010 (BBPR_1707, BBPR_1801 and BBPR_0283) were predicted using the fold recognition method. Taking the structure prediction of BBPR_1707 as an example, the structure prediction procedures are briefly described as follows. To obtain the structure template, we first submitted the sequence of BBPR_1707 to the protein structure prediction metaserver (http://meta.bioinfo.pl/submit_wizard.pl), which integrated a series of well-established fold recognition methods. Using the top hit ranked by the metaserver, the *Corynebacterium diphtheriae* major pilin subunit (SpaA; PDB code: 3HR6; X-ray resolution: 1.6 Å) was chosen as the structure template, which was based on the fold recognition result of the FFAS algorithm [27]. Then, the corresponding sequence alignment between BBPR_1707 and 3HR6 was also obtained from the FFAS algorithm. Finally, the 3D model of BBPR_1707 was generated and refined by the SCWRL program [28]. Similarly, the structures of BBPR_1801 and BBPR_0283 were predicted based on the same template used for BBPR_1707.

### RNA isolation

Total RNA was isolated using the methods described previously [29]. Briefly, cell pellets were resuspended in 1 ml of QUIAZOL (Quiagen, UK) and placed in a tube containing 0.8 g of glass beads (diameter, 106 μm; Sigma). The cells were lysed by shaking the mix on a BioSpec homogenizer at 4°C for 2 min (maximum setting). The mixture was then centrifuged at 12,000 rpm for 15 min, and the upper phase containing the RNA-containing sample was recovered. The RNA sample was further purified by phenol extraction and ethanol precipitation according to an established method [30]*.*

### Reverse transcription -PCR analysis

Five micrograms of mRNA was treated with DNase (Roche, United Kingdom) and used as template in a 100 μl reaction mixture containing 20 ng of random primers, each deoxyribonucleoside triphosphate at a concentration of 0.125 mM, and Superscript enzyme (Invitrogen, Paisley, United Kingdom) used according the manufacturer’s instructions to produce cDNA. The cDNA generated was then used as a template for reverse transcription (RT)-PCRs to determine the arrangement of the transcript encompassing the *pil* loci of *B. bifidum* PRL2010 using the primers listed in Additional file 1.

### Quantitative real-time reverse transcription PCR (qRT-PCR)

**Figure 1:**
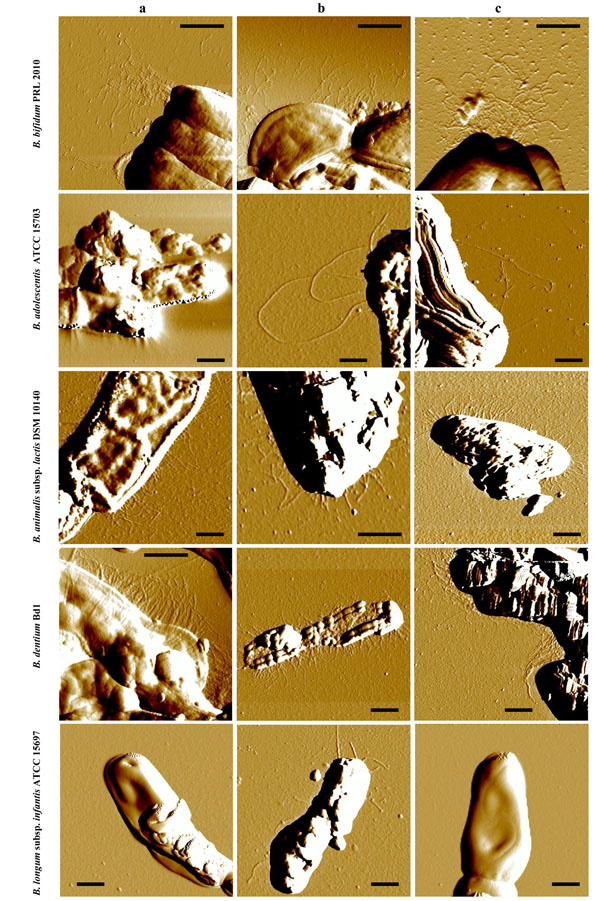
Presence and morphology of pilus-like structures in various bifidobacterial species. Samples were viewed by Atomic Force Microscope**.** Panels a, b and c show pilus-like structures of bifidobacteria cultivated on glucose, lactose or FOS, respectively, as the sole carbon source. Scale bar 0.5 µm.

qRT-PCR primers (Additional file 2) were used to amplify the genes encompassing the *pil* loci as indicated in Figure 1, and the reference genes *atp*D, *tuf*A, *rpo*B and *ldh*. Criteria for primer design were based on a desired melting temperature ™ values between 58 and 60°C and amplicon size of approximately 100 base pairs. qRT-PCR was performed using the CFX96 system (BioRad, CA, USA), fold change was evaluated through the estimation of the CT values with the aid of the CFX96 software (BioRad, CA, USA). PCR products were detected with SYBR Green fluorescent dye and amplified according to the following protocol: one cycle of 95°C for 3 minutes, followed by 39 cycles of 95°C for 5 s and 60°C for 20 s. Melting curve: 65°C to 95°C with increments of 0.5°C/s.

Each PCR reaction mix contained the following: 12.5 μl 2x SYBR SuperMix Green (BioRad, CA, USA), 1 μl of cDNA dilution, each of the forward and reverse primers at 0.5 µM and nuclease-free water was added to obtain a final volume of 20 μl. In each run, negative controls (no cDNA) for each primer set were included.

Fold change was calculated using the CFX96 software (BioRad

## Results and discussion

### Cell morphology and pili structures

The cell surface of bifidobacterial cells belonging to different species spanning a variety of ecological origins (Table 1) was analyzed by Atomic Force Microscopy (AFM). Bacterial cells were cultivated on various carbon sources, including simple carbohydrates, i.e. glucose and lactose, as well as more complex sugars such as fructo-oligosaccharides (FOS), representing those that are expected to be present in the human gut [31]. Such analyses revealed the presence of pilus-like structures on the cell surfaces of various bifidobacteria (Fig. 1). Interestingly, in a couple of cases, such as *B. bifidum* PRL2010 the cell surface was shown to be densely piliated with the majority of pili located at cell-polar position. In contrast, under the conditions examined pilus-like structures were shown to be rare if at all present in *B. longum* subsp. *infantis* ATCC15696 (Fig. 1). When we investigated the production of pilus-like structures in several bifidobacteria propagated under various culture conditions, i.e., growth on different carbon sources, by AFM, we observed differential behavior among the strain/species tested (Fig. 1). Notably, in the case of *B. bifidum* PRL2010 cultivation in MRS medium supplemented with FOS caused a high production of pilus-like structures (Fig. 1), which were often seen as polar tufts. In contrast, a different response, i.e., a less abundant production of pili, to FOS cultivation was noticed on the cell surface of *B. dentium* Bd1. The observed differential phenotypes of *B. bifidum* PRL2010 versus *B. dentium* Bd1 may be linked to the different ecological niches occupied by these micro organisms, represented by the human intestine and oral cavity, respectively.

**Table 1:** Strains used in this study, their origin and effects of the different growth conditions on the on pilus gene/protein expression.

Organism	Origin	Pilus gene cluster	Carbon sources											
			
			Glucose*		lactose		FOS		mucin		Bovine milk		N-acetylglucosamine	
			
			AFM	qRT-PCR	AFM	qRT-PCR	AFM	qRT-PCR	AFM	qRT-PCR	AFM	qRT-PCR	AFM	qRT-PCR
*B. bifidum* PRL2010	Feces of breast-fed infant	282-284		nd		nd		nd		nd		yes		nd
		1707-1709	+	nd	+	nd	+	yes	-	nd	-	nd	-	nd
		1820-1822		nd		nd		nd		nd		yes		nd

*B. longum* subsp. *infantis* ATCC15697	Intestine of infant	-	nd	-	+		nd		-		-		-	

*B. dentium* Bd1	Dental caries	142-144		nd		nd		nd		nd		nd		nd
		197-200		nd		nd		nd		nd		nd		nd
		276-278		-		-		-		-		-		-
		534-536	+	-	+	-	nd	-	-	-	-	-	-	-
		1874-1876		-		-		-		-		-		-
		2000-2002		nd		yes		nd		nd		nd		nd
		2188-2191		-		-		-		-		-		-

*B. animalis* subsp. *lactis* DSM10140	Yogurt	1488-1486	+	nd	+	+	+	+	-	nd	-	nd	-	nd

*B. adolescentis* ATCC15703	Intestine of adult	1467-1470	nd	nd	+	nd	+	+	-	nd	-	+	-	+

*Glucose was used as reference condition for normalizing the qRT-PCR experiments; nd, not identified; +, identified ; -, not performed or present in the genome; ATCC, American Type Culture Collection; DSM, Deutsche Sammlung von Mikroorganismen

The observed sizes of these identified pilus-like structures appeared to be highly variable among the different bifidobacterial species analysed, as well as within the same cell. In particular the length of the filaments was ranging from 100 nm to several micrometers, the width from 10 to 30 nm and the height from 0.5 to 2 nm (Additional file 3). Although the width and the height of these biological structures are affected by the AFM tip size and compression, these measurements indicate the existence of a variable number of pilus-types within the genus *Bifidobacterium*.

### Sequence analysis of bifidobacterial pili-loci

**Figure 2:**
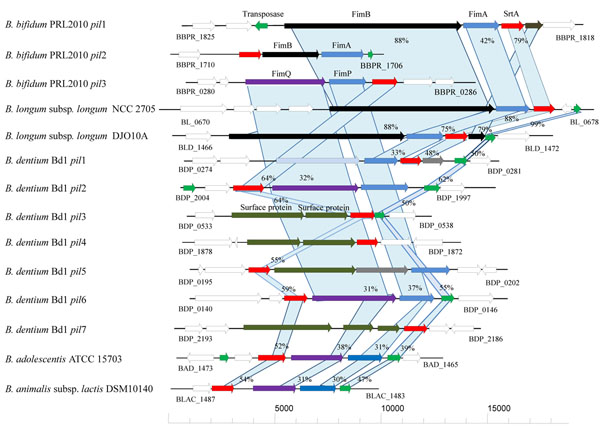
Schematic comparative representation of pilus-encoding loci of various bifidobacterial strains. Each arrow indicates an ORF, the size of which is proportional to the length of the arrow. Colouring of the arrows represents the different function of the gene as indicated indicated above each arrow. The amino acid identity of the relevant encoded proteins is indicated in percentages.

As previously described [1,32,33] pilus-encoding gene clusters in genomes of Gram positive bacteria consist of one to three genes specifying pilus subunits and an associated sortase-encoding gene and can thus be identified based on sequence similarities. Recently, the genome sequences of *B. dentium* Bd1, *B. longum* subsp. *infantis* ATCC 15697, *Bifidobacterium longum* subsp. *longum* NCC2705**,***Bifidobacterium longum* subsp. *longum* DJO10A, *Bifidobacterium bifidum* PRL2010, *Bifidobacterium adolescentis* ATCC15703, and *Bifidobacterium animalis* subsp. *lactis* DSM10140 genomes [19-24] have become available for such purposes. Screening for pilin subunit- and sortase-encoding genes in these genomes revealed the presence of putative pilus gene clusters in all of these genomes with the exception of *B. longum* subsp. *infantis* ATCC15697 (Fig. 2). These putative pilus genetic loci occupy various genomic positions (name designations and genome positions are indicated in Figure 2). Notably, the largest number of pilus gene clusters was identified in the genome of *B. dentium* Bd1, which suggests that this microorganism possesses expanded capabilities to adapt to different ecological environments (e.g., oral cavity as well as intestine and fecal material).

The typical predicted pilus gene cluster identified in these bifidobacterial genomes encompasses genes encoding a major pilin subunit (similar to FimA or FimP) and one or two ancillary minor pilin subunits (similar to FimB and FimQ), which were identified on the basis of amino acid identity with pilin sununits identified in other Gram positive bacteria such as *Actinomyces* spp. and streptoccocae. Furthermore, the sequences of the predicted *fim*AB, *fim*PQ gene products contained the anticipated consensus motifs and domains characteristic of a pilin primary structure, including a Sec-dependent secretion signal, the sortase recognition site (CWSS motif), the pilin-like motif (TVXXK) and the E box (Table 2) [1,3]. Interestingly, all the identified bifidobacterial pilus gene clusters are flanked by transposon elements, indicative of their acquisition by horizontal gene transfer (HGT) (Fig. 2 and see below).

**Table 2:** Genetic features of the major pilin protein in bifidobacteria.

Strain	Major pilin protein			
	ORF	CWSS	Pilin motif	E box
*B. bifidum* PRL2010	283	**LP**K**TG**A	GDSTAE**V**DM**K**	YTVT**ET**AVAD**G**Y
*B. bifidum* PRL2010	1707	**LP**L**TG**G	VYSSGSIDM**K**	YTIE**EI**AAPN**G**Y
*B. bifidum* PRL2010	1821	**LP**G**TG**G	HSTTVG**V**DI**K**	YTLT**ET**EAPA**G**Y
*B.dentium* Bd1	144	**LP**L**TG**A	NHGDNT**V**NM**K**	YTVS**ET**KVAT**G**Q
*B.dentium* Bd1	200	**LP**L**TG**G	QTLSYN**V**TA**K**	YTVK**ET**KAPA**G**Y
*B.dentium* Bd1	277	**LP**E**TG**G	VAGN**V**TITP**K**	YVLT**ET**KTPA**G**Y
*B.dentium* Bd1	535	**LP**L**TG**A	GQTLGV**V**NV**K**	YDVV**ET**DAPA**G**Y
*B.dentium* Bd1	1875	**LP**I**TG**A	HPAQTIDVK**K**	YTVT**ET**VVPA**G**F
*B.dentium* Bd1	2190	**LP**S**TG**G	FEKINS**V**KV**K**	YVLS**ET**KTPE**G**Y
*B.dentium* Bd1	1999	**LP**L**TG**A	PLTLGT**V**VA**K**	YTVK**ET**ATREDL
*B. longum* subsp. *longum* NCC2705	675	**LP**D**TG**G	KSEYPT**V**DKT	YYLK**ET**FAPK**G**Y
*B. longum* subsp. *longum* DJ010A	1468	**LP**G**TG**G	KGSLPT**V**DK**K**	YTLT**ET**KAPA**G**Y
*B. adolescentis* ATCC 15703	1463	**LP**L**TG**A	INAVGMFVA**K**	YTLK**ET**GFAS**G**Y
*B. animalis* subsp. *lactis* DSM10140	1484	**LP**L**TG**A	KPSGTITLG**K**	YKVT**ET**DVLSRY
*A. naeslundii* T14V	FimP	**LP**L**TG**A	WNYNVH**V**YP**K**	YCLV**ET**KAPE**G**Y
*A. naeslundii* T14V	FimA	**LP**L**TG**A	WIYDVH**V**YP**K**	YVLV**ET**KAPA**G**Y
				
Consensus sequences		**LP**x**TG**	xxxxxTVxx**K**	Yxxx**ET**xAPx**G**Y

Notably, in the genome of PRL2010 the ORF1822, encoding a putative FimB, appears to be a pseudogene due to a frame-shift within a stretch of nine guanine residues. However, it is possible that transcriptional slippage along the guanine residues allows expression of this fimbrial subunit under certain environmental conditions [34].

### Structural investigation of pili-like proteins encoded by bifidobacteria

**Figure 3:**
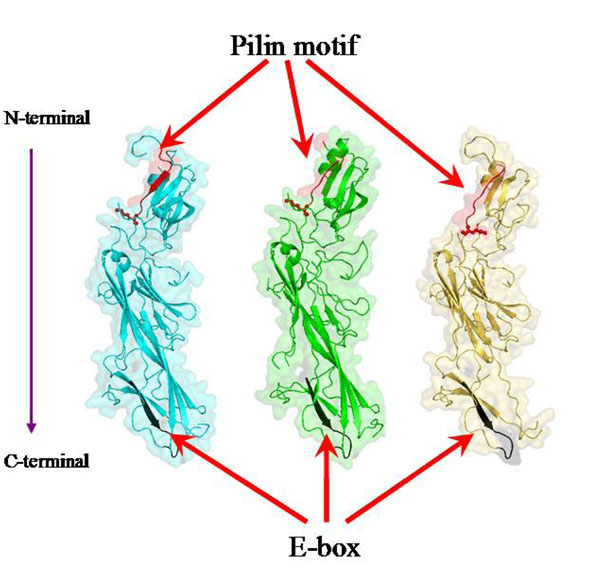
Structural representation of the major pilin subunits of *B. bifidum* PRL2010. BBPR_1707, BBPR_1821 and BBPR_0283 are colored in cyan, green and yellow, respectively. The pilin motifs (residues 188-197 in BBPR_1707, 184-191 in BBPR_1821 and 224-231 in BBPR_0283) are highlighted in red. The side chains of the conserved lysines in these three pilin motifs (K197 in BBPR_1707, K191 in BBPR_1821 and K231 in BBPR_0283) are shown as sticks and balls. The E-box motif in each model is colored in black (residues 445-456 in BBPR_1707, 428-439 in BBPR_1821 and 438-449 in BBPR_0283).

In order to corroborate these findings a structural investigation of *B. bifidum* PRL2010 major subunits encoded by the three *pil* loci present in its genome, was performed. The 3D structures of major pilin subunits (BBPR_1707, BBPR_1821 and BBPR_0283) were predicted based on the *Corynebacterium diphtheriae* major pilin subunit (SpaA, PDB code: 3HR6; X-ray resolution: 1.6 Å) through the FFAS03 program [27]. Similar to the 3D structure of 3HR6 [35], the predicted 3D model also comprises three tandem Ig-like domains. The 3D models of BBPR_1707, BBPR_1821 and BBPR_0283 cover residues of their original sequences from 36-487, 89-470 and 143-467, respectively (Fig. 3). Although the overall protein sequence identity between the three major pili subunits and 3HR6 is only in a range of 22–25 %, the results from fold recognition revealed that the predicted structures of the three major pilin subunits are similar to the 3D structure of 3HR6, which contains three tandem Ig-like domains [35].

In addition, in order to model the general structure of the three major bifidobacterial pilin subunits, the predicted 3D models allow the spatial location of some important functional motifs. As reported previously, the threonine-glycine bond within the LPXTG motif (CWSS motif), which is located at the C-terminal of pilin subunit, is recognized and cleaved by a dedicated sortase. Following this cleavage, the inter-molecular covalent linkage between the threonine of the cleaved LPXTG motif in one pilin subunit and a conserved lysine present within the pilin motif of its neighboring pilin subunit leads to fiber assembly by covalent linkages [36]. Additionally, the glutamic acid within the E-box of the major subunit has been reported to be involved in the incorporation of the minor subunit into the pilus fiber. Based on the predicted 3D models, the corresponding pilin motifs as well as the conserved lysines in the three major pilin subunits can be annotated (Fig. 3). Moreover, the E-box motifs in these three major pilin subunits were also identified (Fig. 3).

Polymerization of the pilin subunits is catalysed by an enzyme with homology to sortases [37]. Notably, all the detected pili genetic loci encompass a sortase encoding gene (*str*A) belonging to the class C family (Fig. 2). Furthermore, genome analyses revealed the presence of a variable number of additional sortases belonging to the housekeeping sortases, also referred as the class A (data not shown). The class A sortases are usually encoded by genes whose chromosomal locations are unlinked to the genes that encode their specific surface protein substrates [38]. These housekeeping sortases might exert an accessory role in the assembly of pili in bifidobacteria, similar to their role in *C. diphtheriae*, where housekeeping sortases are required for the correct covalent attachment of the major pilin subunits to the cell wall [38].

### Phylogenetic analyses of bifidobacterial pilus-associated genes

**Figure 4:**
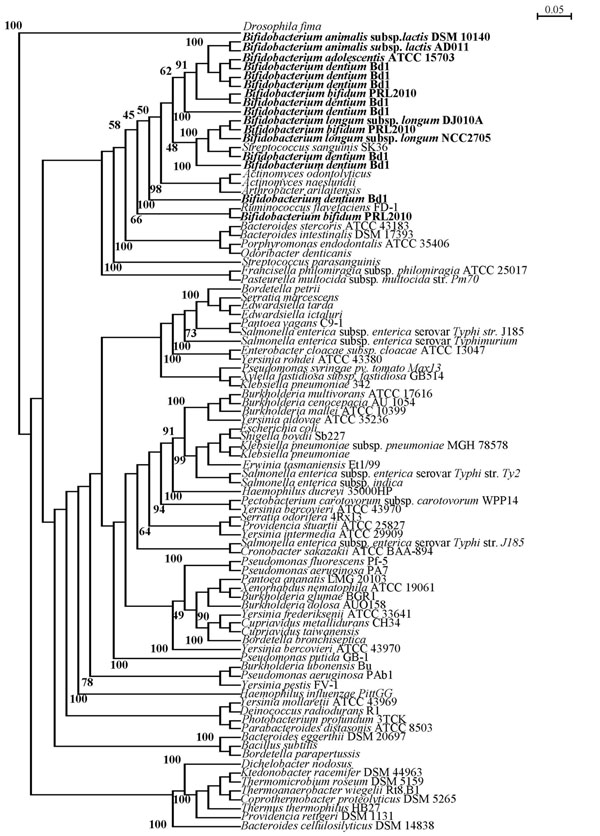
Phylogenetic tree obtained using presumed FimA/P homologs from various bacteria. Bar scale indicates phylogenetic distances. The FimA/P protein sequences from bifidobacteria are typed in bold.

It has been previously shown that pilus-encoding genes in other microorganisms such as corynebacteria have been acquired through Horizontal Gene Transfer (HGT) events [7,39]. We therefore investigated if this may also have been the case for the pilus-encoding genes of bifidobacteria.. In order to assess the distribution of *fim*A/P homologs across bacteria we surveyed currently available genomic data, representing members of *Actinobacteria*, *Firmicutes* and various Gram negative bacteria (Additional file 4) for the presence of genes specifying predicted major pilus subunits. This analysis revealed that predicted FimA/P-encoding genes are not uniformly present in all bacteria studied to date (data not shown). The distribution of *fim*A/P homologs might be a consequence of the organism’s development following a vertical- or horizontal- evolution mechanism. Alignment of the presumed FimA/P-encoding genes was performed using ClustalW and resulted in a rooted neighbour joining phylogenetic tree (Fig. 4). Notably, bifidobacterial FimA/P does form a monophyletic group with other high G+C Gram positive bacteria such as *Actinomyces* spp., as well as with the FimA/P sequences of *Firmicutes* (Fig. 4). Furthermore, bifidobacterial FimA/P sequences cluster together with homologs sequences from Gram negative bacteria such as *Bacteroidetes* spp. (Fig. 4). These phylogenetic inconsistencies based on FimA/P sequences, can be explained by assuming that the bifidobacterial FimA/P-encoding genes, similar to *fimA/P* genes from other high G+C Gram positive, were acquired through HGT events. Other findings that support this hypothesis are the deviating G+C% content of the bifidobacterial *fim*A/P genes compared to the average G+C% content of their genome [e.g. between 5 % lower (in case of *B. longum* subsp. *longum* DJO10A) to 9 % higher (in the case of *B. bifidum* PRL2010 *pil*1) than the G+C average value], as well as by the different codon usage bias for the predicted FimA/P proteins (results not shown).

### Transcriptional analysis of bifidobacterial pili loci

**Figure 5:**
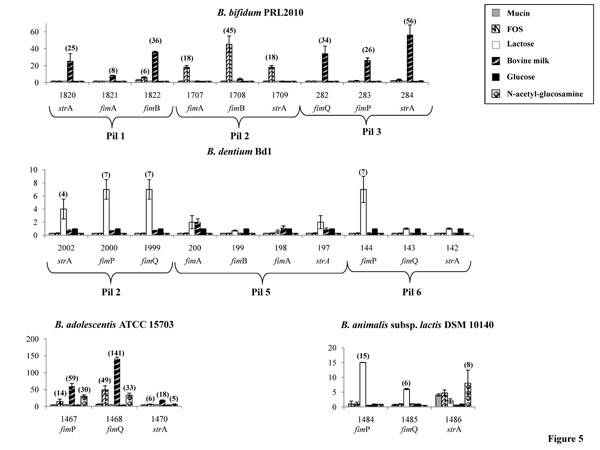
Relative transcription levels of pilus-encoding genes from *B. bifidum* PRL2010, *B. adolescentis* ATCC15703, *B. dentium* Bd1 and *B. animalis* subsp. *lactis* DSM10140 upon cultivation in MRS supplemented with various carbon sources (lactose, FOS, N-acetylglucosamine, mucin), or bovine milk, versus growth in MRS supplemented with glucose as unique carbon source as analysed by quantitative real-time PCR assays. The histograms indicate the relative amounts of the pilin/sortase mRNAs for the specific samples. In each panel, the ORF numbering indicates the gene code according to Figure 1. The values on the y-axes as well as the value (between brackets) represent the fold fold change relative to reference genes as indicated in the materials and method section. For *B. dentium* Bd1 qRT-PCR experiments were performed only on those pili-encoding loci, i.e., *pil*2, *pil*5 and *pil*6, which display a typical gene constellation of the pilus locus (see Figure 2).

In order to determine if the genes encompassing the pili loci detected in bifidobacteria are differentially transcribed upon exposure to different growth substrates simulating those which may be encountered by bifidobacteria in their natural ecological niche, the amount of predicted pilin gene-specific mRNAs was determined by quantitative real-time PCR (qRT-PCR) assays. Such experiments were performed using mRNA samples extracted from exponentially grown cultures of *B. longum* subsp. *longum* DJ010A, *B. bifidum* PRL2010, *B. animalis* subsp. *lactis* DSM10140, *B. adolescentis* ATCC15703 and *B. dentium* Bd1, which had been resuspended in pre-warmed MRS medium containing one of a varied set of growth substrates [lactose, fructo-oligosaccharides (FOS), bovine milk, N-acetyl glucosamine and mucin]. The observed induction level of putative pilus subunit/pilin- or sortase-encoding genes in response to these growth substrates was shown to be variable between the different bifidobacterial species and highly variable between different pilus gene clusters harboured by the same organism (Fig. 5). In *B. adolescentis* ATCC15703 growth on any of these carbohydrate substrates produced an increase of pilin/sortase gene transcription relative to glucose, with the highest level of transcription observed when this strain was cultivated on bovine milk, FOS, or N-acetyl glucosamine (Fig. 5). In contrast, in another intestinal bifidobacterial strain, *B. bifidum* PRL2010, transcription of the putative pilin/sortase-encoding genes was significantly increased relative to glucose-based growth upon cultivation in bovine milk or FOS (Fig. 5). Conversely, the level of pilin-encompassing mRNA of *B. dentium* Bd1, as well as *B. animalis* subsp. *lactis* DSM10140 did not change significantly when this strain was cultivated on any of these substrates (Fig. 5). However, the expression level of pilus-encoding genes in *B. dentium* Bd1 and *B. animalis* subsp. *lactis* DSM10140 changes significantly when such microorganisms were cultivated in the presence of lactose.

The observed differential transcription patterns of the predicted pilus-encoding genes in these bifidobacterial species, i.e., *B. bifidum*, *B. animalis* subsp. *lactis*, *B. adolescentis* and *B. dentium*, may be linked to the specific ecological niche that each of these bacteria occupy (intestine vs. oral cavity as in the case of *B. adolescentis*, *B. bifidum* vs. *B. dentium*, or different niches within the colon, i.e. human intestine vs. animal intestine, such as in the case of *B. bifidum*, *B. adolescentis* vs. *B. animalis* subsp. *lactis*) [15] and thus may be pivotal for their specific colonization strategy. The different pilus gene clusters carried by the genome of a single microorganism (e.g., the *pil*1, *pil*2 and *pil*3 of *B. bifidum* PRL2010) appear to undergo varying levels of transcriptional induction in response to a particular carbohydrate (Fig. 5), which may be explained by the existence of alternative genetic strategies for bacterial colonization and/or microbe-microbe interactions evolved by a specific bifidobacterial strain, similar to what was previously described for *Actinomyces*[8,40]. Furthermore, such findings suggest that these pili promote adhesion to different molecules (e.g., mucin, epithelial cells, enamel).

### Operon organization of the pilus-gene clusters in bifidobacteria

**Figure 6:**
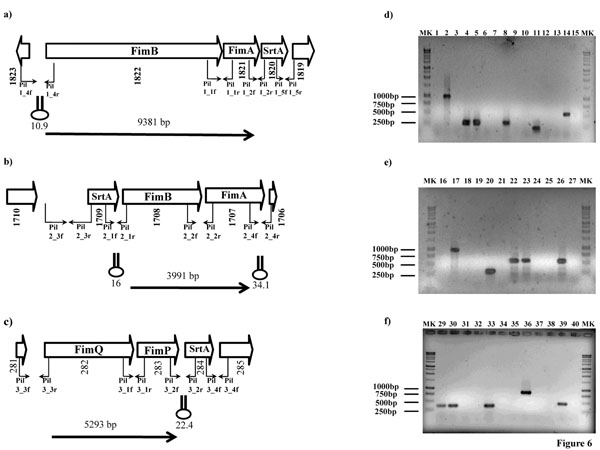
Transcriptional organization of the bifidobacterial pili loci in *B. bifidum* PRL2010. (panel **a-c**) Schematic diagram of bifidobacterial pilus locus. Hairpin symbols indicate predicted secondary structures. Panels **d-f**, products generated by RT-PCR. PCR products were obtained with primers spanning the intergenic regions between the pilin encoding subunits (primers Pil1_1f/ Pil1_1r, Pil2_2f/Pil2_2r and Pil3_1f/Pil3_1r) (lanes 4-5, 22-24, 29-31), between pilin encoding subunits and the *srt*A gene (primers Pil1_2f/Pil1_2r, Pil2_1f/Pil2_1r and Pil3_2f/Pil3_2r) (lanes 7-8, 19-21, 32-34), between pilin encoding subunits and ORF1823, ORF1706, ORF281 (primers Pil1_4f/Pil1_4r; Pil2_4f/Pil2_4r and Pil3_3f/Pil3_3r) (lanes 1-3, 16-18, 35-37), and between the *srt*A gene and ORF 1819, 1710 and 285 (primers Pil1_5f/Pil1_5r; Pil2_3f/Pil2_3r and Pil3_4f/Pil3_4f) (lanes 10-12, 25-27, 38-40). The positions of the primer pairs used in RT-PCR experiments are shown in panels a-c. Products of the PCR were derived under the following conditions: cDNA prepared from *B. bifidum* PRL2010 RNA cultivated on MRS supplemented with bovine milk and/or FOS (lanes 1, 4, 7, 10, 13, 16, 19, 22, 25, 29, 32, 35, 38). A positive control control *B. bifidum* PRL2010 DNA (lanes 2, 5, 8, 11, 14, 17, 20, 23, 26, 30, 33, 36, 39). A negative control in which *B. bifidum* PRL2010 RNA was used but reverse transcriptase was omitted (lanes 3, 6, 9, 12, 15, 18, 21, 24, 27, 31, 34, 37, 40). Lanes MK contained DNA molecular marker 1kb

The genetic organization of the pili gene clusters identified in the individual genomes of investigated bifidobacterial species suggests that the pilus subunit-encoding genes and the adjacent sortase-specifying gene in each cluster are co-transcribed. To examine this further, we selected the pilus-encoding gene clusters identified in the genome of *B. bifidum* PRL2010 for transcriptional analysis using RT-PCR experiments. cDNA templates were obtained by RT of total *B. bifidum* PRL2010 mRNAs, isolated from stationary growth stage cells that had been cultivated on bovine milk and/or FOS. Each cDNA sample was used as template in various PCR reactions that targeted various parts of the pilus-encoding gene cluster and flanking genes through the use of different combinations of primer pairs (Fig. 6). Notably, amplicons of the expected size were achieved using the internal PCR primer pairs spanning the pilin subunits genes (*fim*A/P and *fim*B/Q). In contrast, no PCR products were obtained, when PCR primers were used that targeted the amplification of the intergenic region between the *str*A and the pilin-encoding genes (Fig. 6). When the same strategy was employed to investigate the co-transcription of the pilus gene locus with the other flanking genes, no RT-PCR products were obtained. Therefore, these data indicate that the genes encompassing the *fim*A/P, *fim*B/Q loci produce a single polycistronic mRNA transcript and are thus organized in an operon, a characteristic of known pilus gene clusters [9,41]. Such findings are also corroborated by the evaluation of the change of mRNA levels of the different genes encompassing the pili-loci obtained by qRT-PCR analysis (Fig.5). Notably, the sortase-encoding gene displayed a different level of induction compared to its neighbouring *fim*A/B/P/Q gene, while in the case where no induction was noticed for these latter genes, the sortase gene sometimes displayed the opposite behaviour (e.g., in the case of the pilus-operon of *B. animalis* subsp. *lactis* DSM10140).

Analysis of the nucleotide sequence of the pili loci revealed that the gene clusters were flanked at their 3’ end by an inverted repeat (ΔG values ranging from -22.4 to 34.1 Kcal) that is expected to function as a rho-independent transcriptional terminator structure (Fig. 6), whereas no such sequences were found between the genes of the presumed pilus subunit-encoding genes.

## Conclusions

Although pili are described in both Gram negative and Gram positive bacteria, very little is so far known about the presence of these extracellular structures in bifidobacteria [15,42]. In this study we provide for the first time visual evidence of the existence of pilus-like structures on the surface of bifidobacterial cells belonging to different species (*B. bifidum*, *B. dentium*, *B. longum* subsp. *longum*, *B. adolescentis* and *B. animalis* subsp. *lactis*). However, the function of pili in bifidobacteria remains still to be elucidated. In close related organisms, such as *C. diphtheriae* and *Actinomyces* spp. pilus fibres are important for bacterial adherence to specific host cells, including epithelial cells, erythrocytes and polymorphonuclear leukocytes [5,14] or for the binding of *Actinomyces* to oral streptococci [43,44]; a scenario which is also found for human gut commensals such as lactobacilli [10,11], therefore suggesting that the adhesive function played by pili in bacteria represents a conserved functional role of these structures.

The past few years have seen dramatic advances in our knowledge on the genetics of probiotic bacteria such as bifidobacteria through genome sequencing of a large number of strains [19-24]. However, the mechanisms based on which such commensals recognize and interact with the human host are still unclear. The identification of possible receptors recognized by pili and the function of these receptors in signaling and host defense mechanisms might provide new insights into the molecular mechanisms of microbe-host interactions. In this study we have shown how the expression of the genes encompassing the putative pilus-encoding loci is largely affected by the different composition of the substrate of cultivation, in particular with respect the presence of host-derived products (e.g., mucin or milk). It has been demonstrated that the corynebacterial minor pilin subunits (SpaC and SpaF) plays a pivotal role as receptor in adhesion of the pili to epithelial cells [14]. Considering that many known adhesive receptors in human cells are represented by carbohydrates [45], it is possible that the presence of specific glycans might trigger the expression of pilin-encoding genes. Bifidobacterial strains that were derived from a different ecological origin (e.g., oral vs. intestinal or human vs. animal) display very different transcription induction patterns of the presumptive pilus-gene clusters upon exposure to glycans, which might be a consequence of the fact that these bifidobacteria are adapted to different ecological niches, where the glycan composition is diverse. Furthermore, the identification of pilus-resembling structures decorating the cell surfaces of bifidobacteria whose genomes do not appear to specify sortase-dependent pili, such as *B. longum* subsp. *infantis* ATCC15697, might suggest the existence of additional types of pilus-like structures in bifidobacteria.

At this point we cannot conclusively state that the observed pilus-like surface appendages are encoded by the clusters of genes described in this study. However, recent investigations about the extracellular proteome of *B. animalis* subsp*. lactis* lead to identification of pilin subunits, which correspond to the *B. animalis* subsp. *lactis* pilus-encoding gene cluster (BLAC_1486-BLAC_1484) described in this study [46].

Most bifidobacteria are poorly, if at all, genetically accessible, and therefore it is currently not feasible to perform those experiments needed to confirm the link between the observed pilus structures and their suspected genetic determinants. Future functional genomics investigations directed to the silencing or mutagenesis of the pilus-encoding genes as well as immuno-EM experiments involving antibodies targeting the different pilin subunit will allow an in depth characterization of the genetics of the pilus-like surface appendages displayed by bifidobacteria.

In addition, further studies will be necessary to evaluate the binding activities of these identified pili and to explore how the various pilus-encoding gene clusters from a single microorganism might be expressed in response to colonization of diverse ecological niches and/or exposure to a different microbiota.

## Competing interests

The authors declare that they have no competing interests.

## Supplementary Material

Additional file 1 Primers used in RT-PCR experiments. Click here for file

Additional file 2 Primers used in qRT-PCR experiments. Click here for file

Additional file 3 Profile plot of pili-like structure obtained from the AFM-height images. The noise in the background is mainly due to polylysine used for coating mica. Click here for file

Additional file 4 List of the pili-encoding genes used for the pyhlogenetic anlyses. Click here for file
