# Supplementary material for: Genetic analysis and morphological identification of pilus-like structures in members of the genus Bifidobacterium
Source: Microb Cell Fact. 2011 Aug 30;10(Suppl 1):S16. doi: 10.1186/1475-2859-10-S1-S16 (PMC3231923; doi:10.1186/1475-2859-10-S1-S16)
Supplement: Additional file 1 — Primers used in RT-PCR experiments. [file 1475-2859-10-S1-S16-S1.pdf]

Supplementary Table 1. Primers used in RT-PCR experiments.

Primer	Sequence (5'-3')
Pil1_1 f	GGTAAGACCGTGAACGCCTA
Pil1_1 r	GCTTTCGTATACGGCTGGAG
Pil1_2 f	CTGGTCATTCTCGGTGGAAT
Pil1_2 r	CAGGGTAACACCGGACTTGT
Pil1_4f	CCGACGAGCTGCTAAACTTG
Pil1_4r	ACCTCGATCTGGATGACCTG
Pil1_5 f	CATACGGCATTGATGGTGAG
Pil1_5r	TAGACCTGCATAGCGGAAGC
Pil2_1 f	GGAGACAGCGGTGAAGTCC
Pil2_1 r	GATTTCACGCCCGAACTATG
Pil2_2 f	CGACCTTGAATGTGTGTCCA
Pil2_2 r	GATGGAGGCATGGACGGTG
Pil2_3 f	CACGAGCAATGAATCGAC
Pil2_3 r	GGCATCGTCGAAGACCATC
Pil2_4 f	ACGTTCTCCACGACCACTTC
Pil2_4 r	AGATACTTCCTCCGCGAACA
Pil3_1 f	CGTGACTGGCTTGTGTATGG
Pil3_1 r	CAGTGTCCACTGTCGTCTCG
Pil3_2 f	AGGTGACCAACGTCAAGTCC
Pil3_2 r	GTTTCGATCCGTGTCTCCTC
Pil3_3 f	GATTTCGGCACTGAAGCAG
Pil3_3 r	TCACGATAAGCATCCCTTCC
Pil3_4 f	CGGATCACCATACGGAGTCT
Pil3_4 r	GTGACGATGGTGGTGGTGT
