# Supplementary material for: Genetic analysis and morphological identification of pilus-like structures in members of the genus Bifidobacterium
Source: Microb Cell Fact. 2011 Aug 30;10(Suppl 1):S16. doi: 10.1186/1475-2859-10-S1-S16 (PMC3231923; doi:10.1186/1475-2859-10-S1-S16)
Supplement: Additional file 2 — Primers used in qRT-PCR experiments. [file 1475-2859-10-S1-S16-S2.pdf]

Supplementary Table 2. Primers used in qRT-PCR experiments.

Primer	Sequence (5'-3')	Targeted ORF/gene	Organism
BLAC1484Fw	CTCCAGCTCAAGAAGGTTGG	BLAC1484	*B. animalis* subsp. *lactis* DSM10140
BLAC1484Rv	ATGGTCTTCCTACTGGACG	BLAC1484	*B. animalis* subsp. *lactis* DSM10140
BLAC1485Fw	CGGTCATCCAGGTCGATACT	BLAC1485	*B. animalis* subsp. *lactis* DSM10140
BLAC1485Rv	CACGGTATATGTGCGGTCAG	BLAC1485	*B. animalis* subsp. *lactis* DSM10140
BLAC1486Fw	GAGAACAAGCTGAGGGATGC	BLAC1486	*B. animalis* subsp. *lactis* DSM10140
BLAC1486Rv	CGTGGTATATGGGCAGGTTC	BLAC1486	*B. animalis* subsp. *lactis* DSM10140
BDP0142Fw	CTCCACTGCGGCACGGTACT	BDP0142	*B. dentium* Bd1
BDP0142Rv	TTGCCGATGAGATGCGTGAA	BDP0142	*B. dentium* Bd1
BDP0143Fw	TTCCGATTCCGAAGCAACCA	BDP0143	*B. dentium* Bd1
BDP0143Rv	CCGTTTCATCAGCGCCATTC	BDP0143	*B. dentium* Bd1
BDP0144Fw	GGAAACTTTTCGCCGGCATC	BDP0144	*B. dentium* Bd1
BDP0144Rv	TCAGGTTGGCGTGTGTCAGC	BDP0144	*B. dentium* Bd1
BDP0197Fw	TCGTCTCCGACGGCTCAATC	BDP0197	*B. dentium* Bd1
BDP0197Rv	GGCCATGTTCACGAGGCTTG	BDP0197	*B. dentium* Bd1
BDP0198Fw	TCGAAGGTCATCGCCTCGTC	BDP0198	*B. dentium* Bd1
BDP0198Rv	CCAACGGGTCGTTGTCGAAC	BDP0198	*B. dentium* Bd1
BDP0199Fw	TTCCAGCCGAAATGCGTTGT	BDP0199	*B. dentium* Bd1
BDP0199Rv	CAACGTTTGCCGGTCTCACC	BDP0199	*B. dentium* Bd1
BDP0200Fw	TCGGCCAGACGCTGTCCTAC	BDP0200	*B. dentium* Bd1
BDP0200Rv	CGGAGACCTTGGTGCCATTG	BDP0200	*B. dentium* Bd1
BDP1999Fw	TCAAGCTGCCGAACATGCTG	BDP1999	*B. dentium* Bd1
BDP1999Rv	CGTTAACCGTCTCGCCATCG	BDP1999	*B. dentium* Bd1
BDP2000Fw	TGCCCTGGGTCTTCTTGCTG	BDP2000	*B. dentium* Bd1
BDP2000Rv	CCTGCAGAAGCCGCTGAAAA	BDP2000	*B. dentium* Bd1
BDP2002Fw	GACACACGATGCCCCGAAAC	BDP2002	*B. dentium* Bd1
BDP2002Rv	ATCGCATCGGCGTGATTGAT	BDP2002	*B. dentium* Bd1

BAD1467Fw	ACGAGAAGGCTTTGTGCAGT	BAD1467	*B. adolescentis* ATCC 15703
BAD1467Rv	TGTACGCGGAGTAAGTGTGC	BAD1467	*B. adolescentis* ATCC 15703
BAD1468Fw	GACCAAGCCAACCAGTTCAT	BAD1468	*B. adolescentis* ATCC 15703
BAD1468Rv	TTGGTGGCCTTGTAGTAGCC	BAD1468	*B. adolescentis* ATCC 15703
BAD1470Rv	GTGCAGGTCATCAACGTCAG	BAD1470	*B. adolescentis* ATCC 15703
BAD1470Fw	TCAAAGTGATGGACGAGACG	BAD1470	*B. adolescentis* ATCC 15703
BBP1707FW	AACGCGCCACTGTCCTACAA	BBP1707	*B. bifidum* PRL2010
BBP1707RV	CCCTTGGCCTTGATGTAGCC	BBP1707	*B. bifidum* PRL2010
BBP1708Fw	CGGATACCGGATCACCATCA	BBP1708	*B. bifidum* PRL2010
BBP1708RV	GTTTCCCTCGTCGTCCTTGG	BBP1708	*B. bifidum* PRL2010
BBP1709Fw	GGCGGAGAACGAATTGAAGG	BBP1709	*B. bifidum* PRL2010
BBP1709Rv	AGACGGAACCCTTCGTCTGC	BBP1709	*B. bifidum* PRL2010
BBP1820Fw	TTGTTGCCGTCTCTTGCACT	BBP1820	*B. bifidum* PRL2010
BBP1820Rv	GGCTCGATATCCGCAGAGTC	BBP1820	*B. bifidum* PRL2010
BBP1821Fw	CTGCGACGTTCACTGGTCTC	BBP1821	*B. bifidum* PRL2010
BBP1821 Rv	TGGTGCTATGAACGCGAACT	BBP1821	*B. bifidum* PRL2010
BBP1822 Fw	GCACTATGCTCGGTGCTGTC	BBP1822	*B. bifidum* PRL2010
BBP1822 Rv	ACGACCACATGGCTGATGAC	BBP1822	*B. bifidum* PRL2010
BBP0282Fw	GCGAACAATGATGGCACCTA	BBP0282	*B. bifidum* PRL2010
BBP0282Rv	GTCGAACACCACGACGATGT	BBP0282	*B. bifidum* PRL2010
BBP0283Fw	CACGGTGGAAAACAACCTGA	BBP0283	*B. bifidum* PRL2010
BBP0283 Rv	GGCGTTGTAGGTGATGGTGA	BBP0283	*B. bifidum* PRL2010
BBP0284Fw	CCCTAGCGCTCCAATACCAG	BBP0284	*B. bifidum* PRL2010
BBP0284Rv	CTGGCGAGTTTTGCGTTGTA	BBP0284	*B. bifidum* PRL2010
BD1- rpoB for	AAGACATCATCGCCACCATCAAGT	*rpo*B	*B. dentium* Bd1
BD1- rpoB rev	ATATCATCGACATCCACACGCAGA	*rpo*B	*B. dentium* Bd1
BD1- atpD rev	GGCCAAGGAACTGCTCGATCTTAC	*atp*D	*B. dentium* Bd1
BD1- atpD for	AACAAGGAGCTGCAGGACATCATC	*atp*D	*B. dentium* Bd1
BD1- ldh for	GATTCACCAATCAAGCCCACAAAG	*ldh*	*B. dentium* Bd1
BD1- ldh rev	ATGTCTTCGAGCACGATCTCACG	*ldh*	*B. dentium* Bd1

BBP_rpoB for	GTGCAGACCGACAGCTTCGAC	*rpo*B	*B. bifidum* PRL2010
BBP_rpoB rev	GAGATCTCGTTGAAGAACTCGTC	*rpo*B	*B. bifidum* PRL2010
BBP_atpD for	CAGAGCCGATCAATGGACGTG	*atp*D	*B. bifidum* PRL2010
BBP_atpD rev	GTGCTGCTCGACCTCAAGCGTGAT	*atp*D	*B. bifidum* PRL2010
BBP_ldh for	CACCATGAACAGGAACAAAGTTG	*ldh*	*B. bifidum* PRL2010
BBP_ldh rev	GAATGATCGATGAGTACGAGCTC	*ldh*	*B. bifidum* PRL2010
BLA0atpDFw	CGGCGAGGAAGACAAGACCA	*atp*D	*B. animalis* subsp. *lactis* DSM10140
BLA0atpDRv	TCTCCTCCGCAGGCACGTAG	*atp*D	*B. animalis* subsp. *lactis* DSM10140
BLArpoBFw	GTGGGCGAGCTGATCCAGAA	*rpo*B	*B. animalis* subsp. *lactis* DSM10140
BLArpoBRv	GATGAGTGACTGCGGCGTGA	*rpo*B	*B. animalis* subsp. *lactis* DSM10140
BLA ldhFw	GGCAAGCACTGCACCGAGTT	*ldh*	*B. animalis* subsp. *lactis* DSM10140
BLA ldhRv	CCATATTCGCCGGTGAGCAG	*ldh*	*B. animalis* subsp. *lactis* DSM10140
BADatpFW	TCGAAACCGGTATCAAGGTC	*atp*D	*B. adolescentis* ATCC 15703
BADatpRv	CGCTGAATCATTTCCTGGAT	*atp*D	*B. adolescentis* ATCC 15703
BADldhFW	CTACTCTCGCTTTCGCTGCT	*ldh*	*B. adolescentis* ATCC 15703
BADldhRv	ATGGTCACGGTCGGATAGAA	*ldh*	*B. adolescentis* ATCC 15703
BADrpoFw	CTTCTCCGATCCGTACTTCG	*rpo*B	*B. adolescentis* ATCC 15703
BADrpoRv	CACGGTCTGGGACTTGATTT	*rpo*B	*B. adolescentis* ATCC 15703
