# Supplementary figures and images for: Genetic analysis and morphological identification of pilus-like structures in members of the genus Bifidobacterium
Source: Microb Cell Fact. 2011 Aug 30;10(Suppl 1):S16. doi: 10.1186/1475-2859-10-S1-S16 (PMC3231923; doi:10.1186/1475-2859-10-S1-S16)

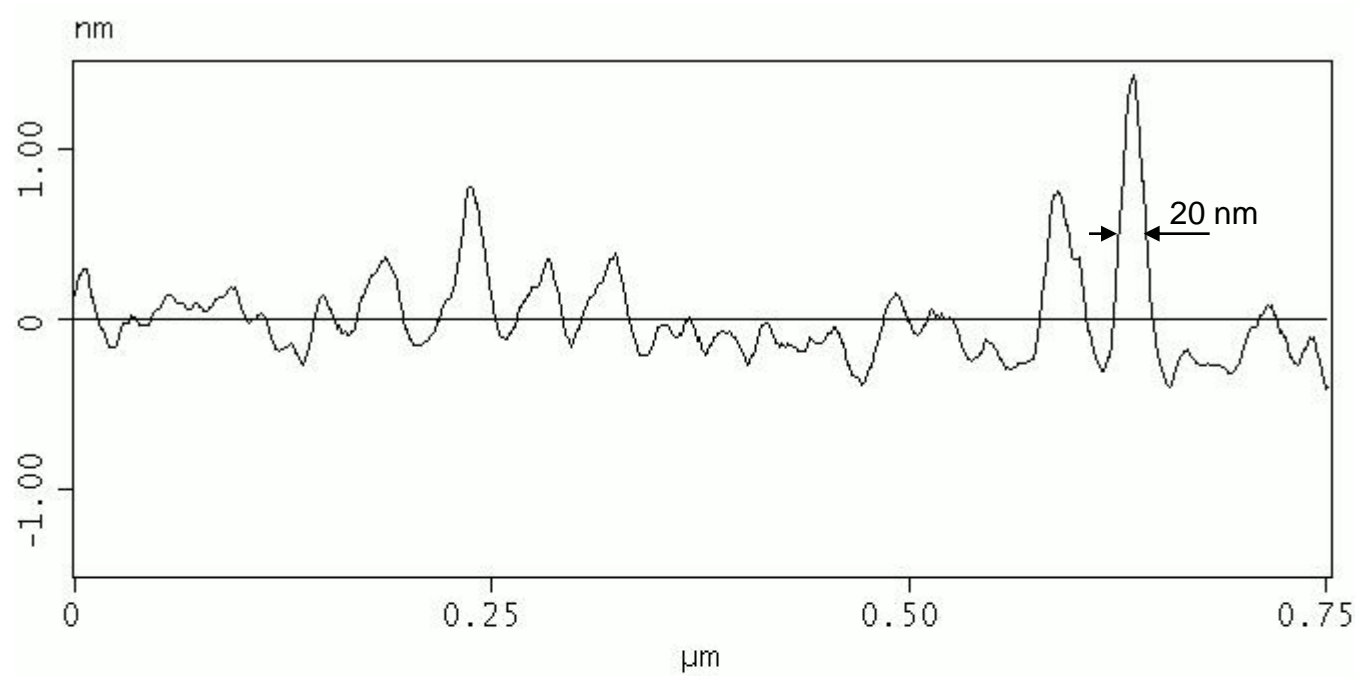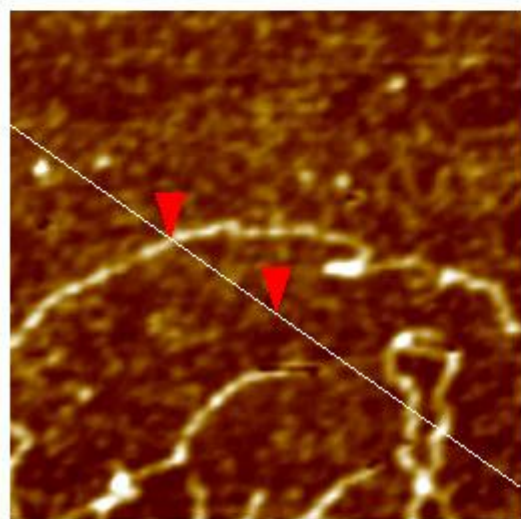

**Supplementary Figure 1**

Supplement: Additional file 3 — Profile plot of pili-like structure obtained from the AFM-height images. The noise in the background is mainly due to polylysine used for coating mica. [file 1475-2859-10-S1-S16-S3.pdf]
