# Supplementary material for: Genetic analysis and morphological identification of pilus-like structures in members of the genus Bifidobacterium
Source: Microb Cell Fact. 2011 Aug 30;10(Suppl 1):S16. doi: 10.1186/1475-2859-10-S1-S16 (PMC3231923; doi:10.1186/1475-2859-10-S1-S16)
Supplement: Additional file 4 — List of the pili-encoding genes used for the pyhlogenetic anlyses. [file 1475-2859-10-S1-S16-S4.pdf]

Supplementary Table 3. List of the pili-encoding genes used for the phylogenetic analyses .

Organism	Gene function	Accession number
Bifidobacterium animalis subsp. *lactis* DSM 10140	Cell surface protein fimA fimbrial subunit	ACS48397.1
Bifidobacterium. animalis subsp. *lactis* AD011	Possible cell surface protein	ACL28972.1
Bifidobacterium. adolescentis ATCC 15703	Cell surface protein fimA fimbrial subunit	YP_910330.1
Bifidobacterium dentium Bd1	Cell surface protein	YP_003361571.1
Bifidobacterium dentium Bd1	Surface protein with Cna protein B-type domain	YP_003360030.1
Bifidobacterium dentium Bd1	Fimbrial subunit-like cell surface protein	YP_003359706.1
Bifidobacterium bifidum PRL2010	Fimbrial subunit *fim*A	YP_003970445.1
Bifidobacterium dentium Bd1	Cell surface protein	YP_003361281.1
Bifidobacterium dentium Bd1	LPXTG cell wall surface protein,	YP_003361282.1
Bifidobacterium longum subsp. *longum* DJO10A	Fimbrial subunit *fim*A	ZP_00121141.2
Bifidobacterium bifidum PRL2010	Cell surface protein	YP_003971869.1
Bifidobacterium longum subsp. *longum* NCC2705	Fimbrial subunit f*im*A	NP_695860.1
Streptococcus sanguinis SK36	*fimA* fimbrial subunit-like protein, putative	ABN45021.1
Bifidobacterium dentium Bd1	Sortase-anchored surface protein	YP_003359781.1
Bifidobacterium dentium Bd1	Fimbrial subunit	YP_003361396.1
Actinomyces odontolyticus	Type-2 fimbrial major subunit precursor	ABD84025.1
Actinomyces naeslundii	Fimbrial structural subunit	AAC13545.1
Arthrobacter arilaitensis Re117	Putative fimbrial structural subunit	CBT74507.1
B. dentium Bd1	Collagen adhesin precursor	YP_003361570.1
Ruminococcus flavefaciens FD-1	Fimbrial subunit *fim*A	ZP_06142946.1
B. bifidum PRL2010	Fimbrial subunit f*im*A	YP_003970445.1
Bacteroides stercoris ATCC 43183	Major fimbrial subunit protein (*fim*A)	ZP_02436541.1
Bacteroides intestinalis DSM 17393	Major fimbrial subunit protein (*fim*A)	EDV04845.1
Porphyromonas endodontalis ATCC 35406	fimbrillin	EEN83125.1
Odoribacter denticanis	Fimbrial subunit *fim*A	AAT76446.1
Streptococcus parasanguinis	Adhesin specific for salivary pellicle of dental surfaces	AAA53077.1
Francisella philomiragia subsp. *philomiragia* ATCC 25017	Fimbrial subunit *fim*A	ABZ87973.1
Pasteurella multocida subsp. *multocida* str. *Pm70*	Fimbrial subunit *fim*A	AAK03010.1
Bordetella petrii	Putative fimbrial adhesin	CAP44477
Serratia marcescens	Fimbrial subunit *fim*A	AAW62392.1
Edwardsiella tarda	Major fimbrial subunit protein	ABK57119.1
Edwardsiella ictaluri	Putative major fimbrial subunit protein	AAT41671.1
Pantoea vagans C9-1	Type-1 fimbrial protein, A chain precursor	YP_003933538.1
Salmonella enterica subsp. *enterica* serovar *Typhi* str. J185	Fimbrial protein	ZP_03380023.1
Salmonella enterica subsp. *enterica* serovar *Typhimurium*	Putative fimbrial subunit A	AAF21713.1
Enterobacter cloacae subsp. cloacae ATCC 13047	P pilus assembly protein, pilin *fim*A	ADF59657.1
Yersinia rohdei ATCC 43380	P pilus assembly protein, pilin *fim*A	EEQ04210.1
Pseudomonas syringae pv. *tomato* Max13	Type I pilus biogensis protein *fim*A	ZP_07232260.1
Xylella fastidiosa subsp. *fastidiosa* GB514	Fimbrial protein	ADN63049.1
Klebsiella pneumoniae 342	Type-1 fimbrial protein	ACI10061
Burkholderia multivorans ATCC 17616	Major type 1 subunit fimbrin	BAG43555.1
Burkholderia cenocepacia AU 1054	Fimbrial protein	ABF76053.1
Burkholderia mallei ATCC 10399	Fimbrial subunit *fim*A	EDP89455.1
Yersinia aldovae ATCC 35236	Fimbrial protein	EEP93769.1
Escherichia coli	*fim*A precursor	CAA85727.1
Shigella boydii Sb227	Major type 1 subunit fimbrin	ABB68780.1
Klebsiella pneumoniae subsp. *pneumoniae* MGH 78578	Major type 1 subunit fimbrin (pilin)	YP_001336915.1
Klebsiella pneumoniae	Fimbrial subunit *fim*A	ACV60168.1
Erwinia tasmaniensis Et1/99	Type-1 fimbrial protein, FimA	CAO97915.1
Salmonella enterica subsp. *enterica* serovar *Typhi* str. Ty2	Type-1 fimbrial protein subunit A	NP_806054.1
Salmonella enterica subsp. *indica*	Major pilin protein *fim*A	AAD23947.1
Haemophilus ducreyi 35000HP	Fimbrial major pilin protein	NP_872871.1
Pectobacterium carotovorum subsp. *carotovorum* WPP14	Fimbriae major subunit protein	ZP_03832453.1
Yersinia bercovieri ATCC 43970	P pilus assembly protein, pilin FimA	EEQ06994.1
Serratia odorifera 4Rx13	Type-1 fimbrial protein, A chain	EFA17402.1
Providencia stuartii ATCC 25827	Pilus assembly protein, pilin f*im*A	EDU57522.1
Yersinia intermedia ATCC 29909	P pilus assembly protein, pilin *fim*A	EEQ19614.1
Salmonella enterica subsp. *enterica* serovar *Typhi* str. J185	Fimbrial subunit	ZP_03379271.1
Cronobacter sakazakii ATCC BAA-894	Pilus assembly protein, pilin *fim*A	ABU76251.1
Pseudomonas fluorescens Pf-5	Type I pilus biogensis protein *fim*A	AAY90745.1
Pseudomonas aeruginosa PA7	Type I pilus biogensis protein *fim*A	ABR83321.1
Pantoea ananatis LMG 20103	Fimbrial subunit *fim*A	ADD76691.1
Xenorhabdus nematophila ATCC 19061	Putative Fimbrial subunit (pilin)	CBJ89795.1
Burkholderia glumae BGR1	P pilus assembly protein, pilin *fim*A	ACR30627.1

Burkholderia dolosa AUO158	P pilus assembly protein pilin *fim*A	EAY67592.1
Yersinia frederiksenii ATCC 33641	P pilus assembly protein, pilin *fim*A	EEQ12942.1
Cupriavidus metallidurans CH34	Fimbrial protein	ABF08548.1
Cupriavidus taiwanensis	Major type 1 subunit fimbrin (pilin)	CAQ72393.1
Bordetella bronchiseptica	Putative major fimbrial structural subunit	AAB94076.1
Yersinia bercovieri ATCC 43970	P pilus assembly protein, pilin FimA	ZP_04627370.1
Pseudomonas putida GB-1	Fimbrial protein	ABY97371.1
Burkholderia ubonensis Bu	Type I pilus biogensis protein FimA	ZP_02379636.1
Pseudomonas aeruginosa PAb1	Putative fimbrial protein	ZP_06881617.1
Yersinia pestis FV-1	Fimbrial protein	ZP_02332403.1
Haemophilus influenzae PittGG	Putative F17-like fimbrial subunit	YP_001293149.1
Yersinia mollaretii ATCC 43969	P pilus assembly protein, pilin FimA	ZP_04639781.1
Deinococcus radiodurans R1	Adhesin B	NP_296243.1
Photobacterium profundum 3TCK	Pilus assembly protein, pilin FimA	EAS44350.1
Parabacteroides distasonis ATCC 8503	Putative fimbrilin precursor	ABR45199.1
Bacteroides eggerthii DSM 20697	Fimbrial subunit *fim*A	EEC55246.1
Bacillus subtilis	YcdI	BAA22247.1
Bordetella parapertussis	Putative major fimbrial structural subunit	AAB94079.1
Dichelobacter nodosus	Fimbrial subunit AC6	AAA23337.1
Ktedonobacter racemifer DSM 44963	Putative peptidoglycan binding domain	EFH87082.1
Thermomicrobium roseum DSM 5159	Major pilin protein f*im*A	ACM06830.1
Thermoanaerobacter wiegelii Rt8.B1	Putative peptidoglycan binding domain	EFN47949.1
Coprothermobacter proteolyticus DSM 5265	Major pilin protein *fim*A	ACI17737.1
Thermus thermophilus HB27	Major pilin protein *fim*A	AAS81862.1
Providencia rettgeri DSM 1131	Major pilin protein *fim*A	ZP_06125206.1
Bacteroides cellulosilyticus DSM 14838	Fimbrial subunit *fim*A	EEF88619.1
Drosophila fima	amylase	AAC47377.1
